# Epidemiology of acquired hypothalamic obesity following traumatic brain injury and nonspecific hypothalamic microinjury: A nationwide German claims data analysis

**DOI:** 10.1111/jne.70108

**Published:** 2025-11-14

**Authors:** Julian Witte, Nicolas Touchot, Manuel Batram, Jana Diekmannshemke, Mathias Flume, Hermann L. Müller

**Affiliations:** ^1^ Vandage GmbH Bielefeld Germany; ^2^ Rhythm Pharmaceuticals, Inc Boston Massachusetts USA; ^3^ Gene Access GmbH Dortmund Germany; ^4^ Department of Pediatrics and Pediatric Hematology/Oncology, University Children's Hospital Carl von Ossietzky University, Klinikum Oldenburg AöR Oldenburg Germany

**Keywords:** acquired hypothalamic obesity, neuroendocrine disease, rapid weight gain, septo‐optic dysplasia, traumatic brain injury

## Abstract

Acquired hypothalamic obesity (aHO) is characterized by rapid and persistent weight gain resulting from structural or functional damage to the hypothalamus, typically accompanied by neuroendocrine dysfunction. While aHO is well described in the context of hypothalamic or suprasellar tumors, particularly craniopharyngioma, little is known about its epidemiology in non‐tumor–related etiologies such as traumatic brain injury (TBI) or subtle, unrecognized hypothalamic injuries. This study estimates the incidence and clinical characteristics of aHO in patients with traumatic brain injury (TBI‐aHO) and hypothalamic–pituitary axis dysfunction of unclear origin, referred to as unspecified microinjury (UM‐aHO). We conducted a retrospective cohort study using German statutory health insurance claims data (*N* = 6.3 million, 2010–2022). Patients were included based on either an incident diagnosis of TBI or a diagnostic algorithm indicating hypothalamic–pituitary axis dysfunction without a documented causal event. aHO was defined via incident obesity coding and validated by concomitant arginine vasopressin deficiency (AVP‐D) and desmopressin prescription. For UM‐aHO, additional validation required at least three concurrent neuroendocrine replacement therapies. Sensitivity analyses assessed the robustness of case definitions. The estimated incidence of TBI‐aHO was 1.78 per million persons (mean age: 42 years; 27% female), corresponding to approximately 520 prevalent cases in Germany. UM‐aHO showed a slightly higher incidence of 2.12 per million (mean age: 37 years; 55% female), with an estimated 660 prevalent cases. Following the diagnosis of incident obesity, most patients in both cohorts received at least two neuroendocrine therapies in addition to desmopressin, most commonly including hydrocortisone and levothyroxine sodium. This is the first population‐based study to characterize aHO following non‐tumor–related hypothalamic injury. Both TBI and subtle, nonspecific hypothalamic microinjuries contribute meaningfully to the burden of aHO in clinical practice. These findings underscore the need for increased clinical awareness and early recognition of hypothalamic dysfunction in patients beyond classical tumor etiologies.

## 1 INTRODUCTION

Hypothalamic obesity (HO) is a serious, heterogeneous disease related to several conditions that lead to damage or dysfunction of the hypothalamus. Acquired HO (aHO) can be defined as an acquired impairment of the melanocortin‐4 receptor (MC4R) pathway due to hypothalamic physical injury or structural abnormality often associated with hypopituitarism, and is characterized mainly by a sudden, abnormal change in weight trajectory that is sustained over time.^1^ Craniopharyngioma is most often associated with aHO in the literature, accounting for ≥50% of all HO cases.^2^, ^3^ Currently, published epidemiological estimates focus on tumor/treatment‐related aHO (TTR‐aHO). The most recent data from Germany^3^ estimate the incidence of TTR‐aHO at 0.7–1.7 cases per million annually, corresponding to a prevalence of 1260 patients. However, because aHO is a consequence of an injury to the hypothalamus, it can be caused by many other tumor‐ and non‐tumor–related conditions.^4^, ^5^, ^6^ These include various forms of injury affecting the hypothalamus or the hypothalamic–pituitary axis, among which are traumatic brain injury (TBI)^7^ or smaller, nonspecific injuries such as viral infections, inflammation, or small vessel occlusion, later defined as unspecified microinjury (UM).^8^, ^9^ Yet, evidence is scarce regarding the frequency of aHO in other underlying conditions.

Research on weight changes after TBI in children and adults reveals complex patterns, although it is well documented that neuroendocrine dysfunctions are common following TBI.^10^, ^11^ While initial weight loss is common due to hypermetabolism and reduced caloric intake,^12^, ^13^ rapid weight gain is common following inpatient TBI treatment.^13^, ^14^ TBI lesions can impair hypothalamic regulation of appetite, which may, in some individuals, contribute to hyperphagia and subsequent weight gain.^13^ However, the true prevalence of post‐TBI weight gain remains largely uncertain, as both the definition and classification of TBI—particularly in terms of severity—vary considerably across studies. A longitudinal analysis reports 42% of patients with TBI gaining weight over time [9] with obesity rates increasing with increasing post‐injury time.^15^ Crenn et al. noted similarity in food‐seeking behavior between patients recovering from TBI and patients with Prader–Willi syndrome.^13^ However, despite the evidence suggesting that TBI can lead to the development of rapid and severe obesity due to hypothalamic damage, aHO remains under researched in this context.

In addition, inflammation, infections, and vascular injury to the hypothalamus, the hypothalamic–pituitary axis, or related structures could also lead to functional impairment of the hypothalamic–pituitary system. Therefore, patients with UM to the hypothalamic–pituitary axis can also be prone to aHO. In contrast to patients with tumors or TBI, for whom the cause of hypothalamic damage is clear, patients with UM may develop clinical signs of hypothalamic damage and aHO without a clearly documented or relatable cause. These patients may have suffered subclinical or unreported injuries, have undiagnosed underlying conditions, or have an otherwise undescribed etiology. Therefore, we define UM as damage to the hypothalamic–pituitary axis, which occurred at an unspecified time, due to unspecified reasons, and which is identifiable by an algorithm of aHO‐related diagnoses and therapies.

This study aims to estimate the distribution and related incidence of patients who develop indicators of aHO following incident TBI or UM. Pharmacological management of other neuroendocrine diseases is further evaluated.

## 2 MATERIALS AND METHODS

### 2.1 Study design and database

We conducted a retrospective cohort study using claims data from January 2009 to December 2022 from German Statutory Health Insurance (SHI), a system covering 88% of the total German population.^16^ GWQ ServicePlus AG, a joint venture of medium‐sized SHI funds in Germany, provided the data. The dataset comprises information on up to 6.3 million people insured at 19 SHI funds, representing up to 8.4% of the total German SHI population. Based on a comparison with official statistics published by the German Health Ministry on SHI (“KM6”), the dataset is representative of the German SHI population in terms of age and gender distribution.^17^

The initial dataset represents all persons in the SHI database aged between 0 and 100 years. Individual information on the birth year was aggregated to 5‐year intervals in the anonymization process. We included patients with a diagnosis of a TBI or a documented diagnosis associated with a UM (for study cohort definitions see Table S1). From January 2010 until December 2020, patients with at least one diagnosis for these underlying conditions were eligible for inclusion, applying a 1‐year washout period (see Figure S1). Identified patients were followed for at least 2 years, allowing for a minimum follow‐up period of 2 years for outcome evaluation. We validate the incidence of the underlying condition by using a 1‐year washout period.

Available data comprise all diagnoses documented during physician outpatient contact and patient hospital stays. German claims data do not include laboratory results, anthropometric data, magnetic resonance imaging (MRI) findings, detailed tumor characteristics, or detailed information on physician contact during inpatient stays. While prescription data are available, prescriptions and their specific indications are not linked directly. A general description of the claims database in the German setting is available from Swart et al.^18^

The study relied on anonymized insurance claims data and was conducted according to general data protection rules and the ethical principles of the Declaration of Helsinki. The study was noninterventional and received positive ethical approval.

### 2.2 Study population

#### 2.2.1 Patient identification

**FIGURE 1:**
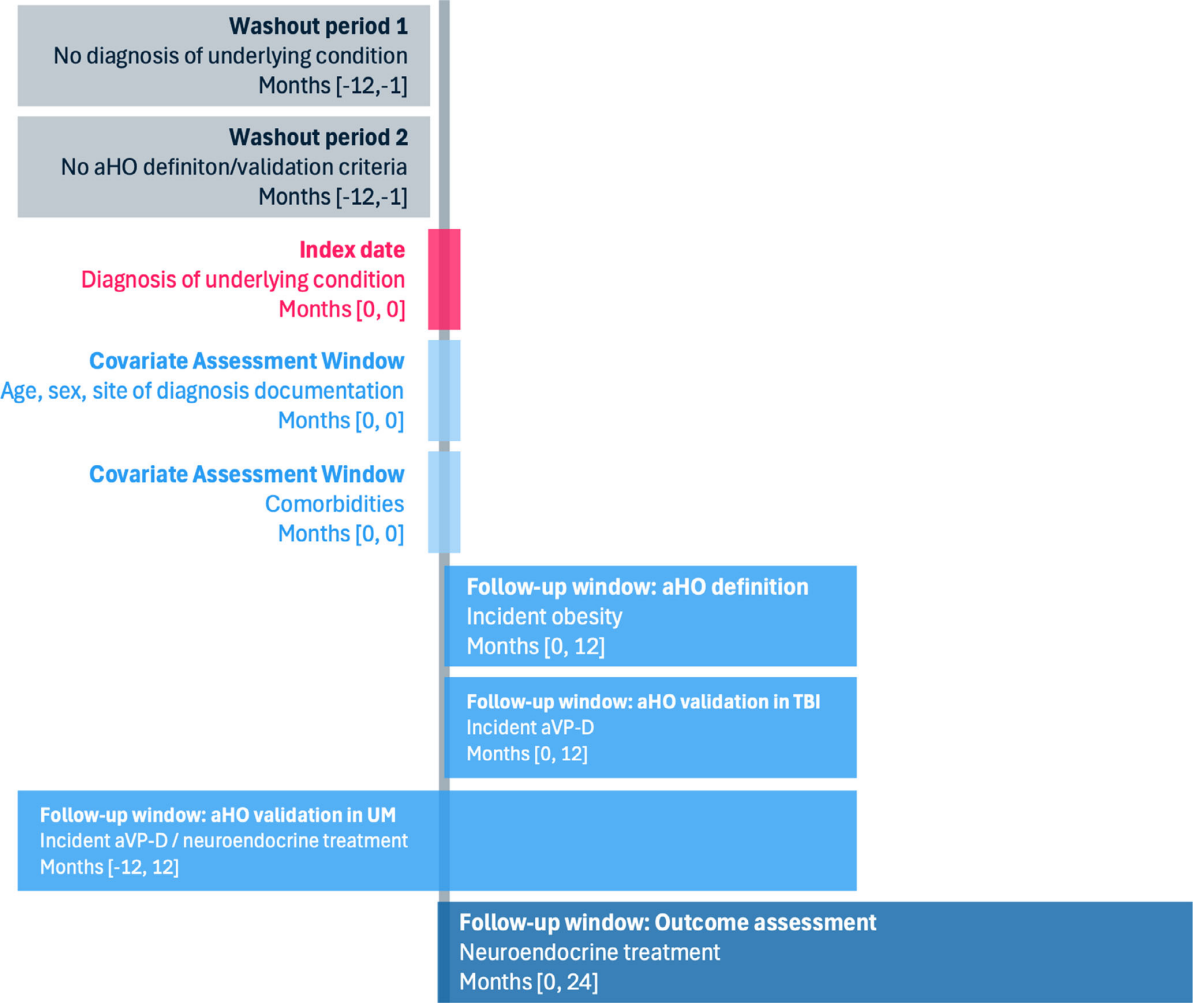
Study schematic of patient inclusion and outcome evaluation. aHO, acquired hypothalamic obesity; AVP‐D, arginine vasopressin deficiency; TBI, traumatic brain injury; UM, unspecified microinjury.

Patient identification is based on the literature and is aligned with medical experts' opinions. Patient inclusion is based on the first identified diagnosis of the respective underlying condition (“index date”) likely related to a hypothalamic injury and aHO (Figure 1). For patients with TBI, the index date is the first diagnosis of TBI and thus related to a specific event indicating the beginning of the potential aHO underlying condition (see Table S1). In contrast, for patients with UM, there is no documentation of a trauma, tumor, or treatment (“events”) known to cause hypothalamic damage. Patients with UM are included based on an algorithm requiring the presence of at least one diagnosis of hypopituitarism, other disorders of the pituitary gland or disorders of the optic nerve, in combination with other criteria related to hormone deficiencies, or neurological symptoms within 2 years thereafter (see Table S1). The index date for patients with UM is the first identified diagnosis related to hypothalamic damage. We also applyed a 1‐year washout period for these patients, to establish structural equivalence within the UM cohort (see Figure S1). However, the index date in this population is not equivalent to an index event as in the TBI cohort.

A one‐time documentation of defined diagnoses in the inpatient setting (primary or secondary diagnosis) or in the outpatient physician setting (only confirmed diagnoses, which excludes so‐called suspected diagnoses) is sufficient for patient inclusion. Analyses may double‐count individuals with multiple conditions being considered; this also applies to previously reported estimates of tumor and/or treatment‐related aHO (TTR‐aHO).^3^ Thus, patients already identified with TTR‐aHO are excluded from the TBI‐aHO and UM‐aHO cohorts after patient identification.

To differentiate between aHO and genetic types of HO, patients with a history of Prader–Willi Syndrome (International Classification of Diseases 10th Revision German Modification [ICD‐10‐GM] Q87.1) are excluded. Other ultra‐rare genetic conditions, such as Bardet–Biedl syndrome, Alström syndrome, or monogenic obesity are not excluded explicitly, as in these conditions, obesity would usually occur in the first years of life and would likely predate the acquired hypothalamic damage used in our identification algorithm.

#### 2.2.2 aHO definition

Lacking patient‐level weight information in German claims data, we applied an incident obesity or rapid weight gain diagnosis (ICD‐10‐GM diagnosis E66.x, E67.x, E68, R63.2) within 12 months after the index date as a mandatory criterion for defining a patient with aHO. Incident obesity is validated based on the preceding 12 months without an obesity diagnosis.

#### 2.2.3 aHO validation

In the absence of an ICD‐10‐GM coding for an operational definition of aHO, we apply arginine vasopressin deficiency (AVP‐D; ICD‐10‐GM diagnosis E23.0, E23.2, E23.3, E23.6, E23.7) and a desmopressin prescription (Anatomical Therapeutic Chemical [ATC] classification code H01BA02) within the post‐observational period as criteria to validate an observed weight gain (approximated by incident obesity diagnosis). The use of AVP‐D as a validation criterion was used successfully to validate aHO within a tumor/treatment cohort in a previous publication by Witte et al.,^19^ as the development of AVP‐D can be considered an endocrine marker of more severe hypothalamic damage.

Since the index event for incident TBI cases in claims data is clearly identifiable, we assume a high sensitivity and specificity of the described identification algorithm. This may not be true for patients with UM, as these injuries could result from chronic conditions that do not necessarily require annual treatments, or could result from another condition, such as a viral infection, which is not overtly associated with the underlying problem of hypothalamic–pituitary dysfunction. We, therefore, apply an additional aHO validation criterion to this population; a three‐fold neuroendocrine replacement therapy must also be initiated 1 year before or after incident obesity. For this, at least three therapies with desmopressin, hydrocortisone, levothyroxine sodium, somatropin, or male and female hormone replacement therapies (e.g., testosterone, estrogen, progesterone, estradiol, estriol; see Table S2 for further details), are required. Distribution of prescriptions for neuroendocrine replacement therapy in patients with aHO‐TBI is also reported but is not part of the TBI‐aHO validation algorithm.

Definition of aHO and other relevant patient identification criteria are made based on literature^8^, ^20^, ^21^ and following the review of medical records of TBI and other aHO patients treated in a large German pediatric clinic with >100 cases of aHO per year. aHO definition and validation criteria can be related to different physician contacts or hospitalizations.

### 2.3 Outcome measures

The primary outcome measure is the distribution of patients with validated aHO in a baseline population with TBI or with UM. aHO incidence rates are further calculated utilizing the full observational period and aHO prevalence rates are extrapolated to the total German population. Results are differentiated by age, sex, and underlying condition. We also describe pharmacological neuroendocrine management post index date.

### 2.4 Statistical analysis

The percentages of patients with incident obesity (aHO definition) who met the validation criteria (aHO validation) are reported by their respective underlying condition. Incidence rates are reported using the midterm population‐at‐risk. Prevalence of aHO was calculated as a function of estimated incidence and survival rate. For patients with TBI, we assumed a mean survival after a TBI event of 30.6 years (95% CI 26.8–34.5). Since we exclude patients who died due to TBI through the 2‐year follow‐up evaluation, we can rely on calculations for the long‐term survival after TBI.^22^ For patients with UM, no reliable assumptions about life expectancy are available; thus we assume the same values as for the cohort with TBI.

We explored uncertainty associated with epidemiological estimates of aHO by varying applied validation criteria: validation of aHO based on patients with prevalent obesity diagnoses before the index date, and validation of aHO without desmopressin prescription. Further, the additional criterion on the number of targeted drug combinations in patients with UM is eased, including patients with desmopressin plus one additional therapy.

Due to data protection constraints, only groups with *n* ≥ 5 could be analyzed and reported. Reporting of study results followed STROBE criteria.^23^ We performed analyses with R (Version 4.1.3), a standard, open‐access, statistical computing programming language.

## 3 RESULTS

### 3.1 Study population

**FIGURE 2:**
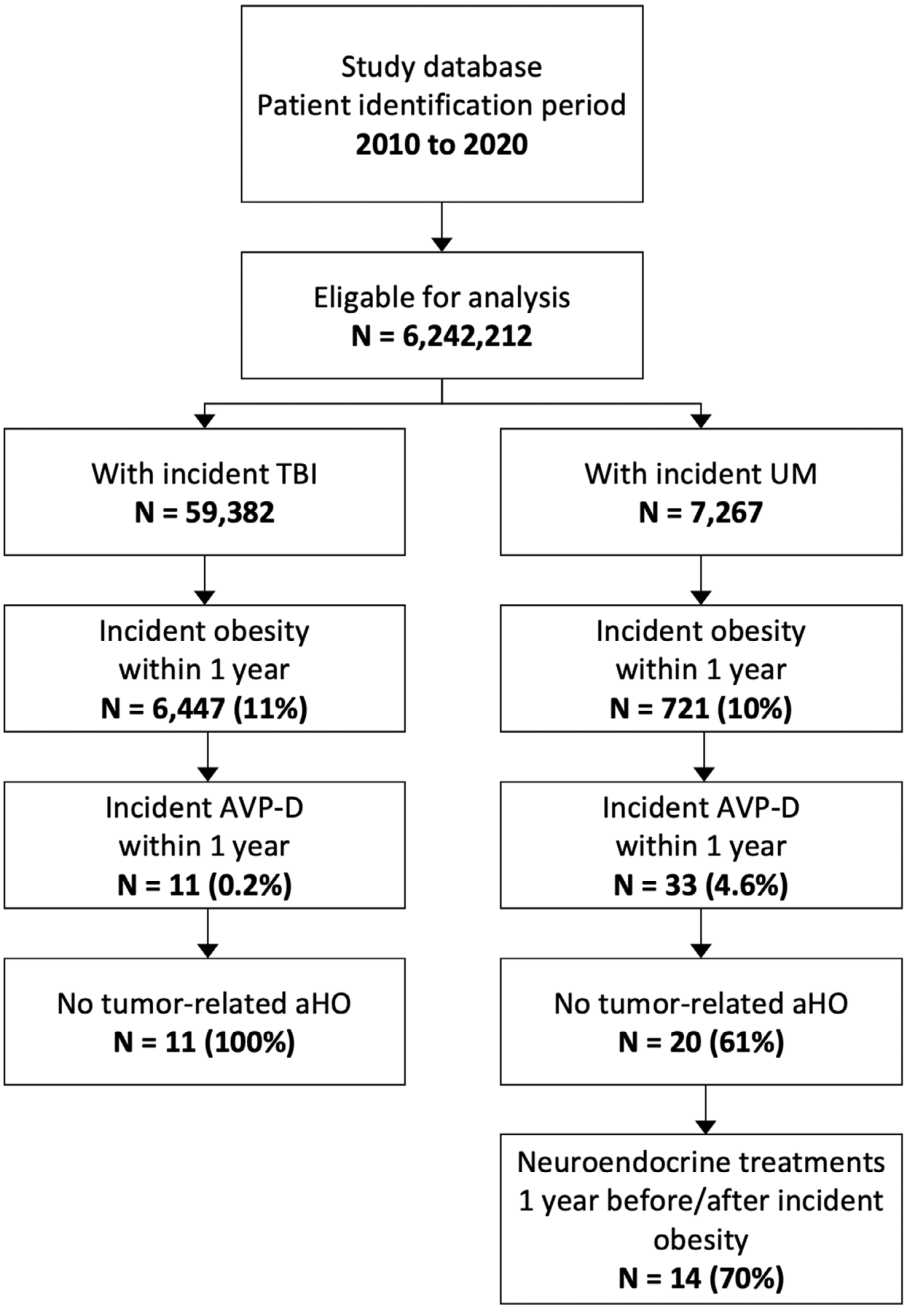
Results of aHO patient identification in patients with TBI and UM. aHO, acquired hypothalamic obesity; AVP‐D, arginine vasopressin deficiency; TBI, traumatic brain injury; UM, unspecified microinjury.

We analyzed a total of 6,242,212 insured persons covered in the study database between 2010 and 2020 (time frame of patient inclusion). Overall, 59,382 persons who met the diagnostic criteria for incident TBI and 7267 persons who met the diagnostic criteria for UM leading to hypothalamic damage were included in the analysis. Of the patients with TBI and UM who develop incident obesity within 1 year after the index date, 0.2% and 4.6%, respectively, also met the criteria for AVP‐D (Figure 2).

### 3.2 aHO in patients with TBI

Among all patients with TBI identified in the study dataset, 11 developed a validated aHO without having a tumor etiology. These patients are, on average, 42 years old, and around 25% are female (Table 1). The estimated TBI‐aHO incidence is 1.8 cases per 1,000,000 persons. Extrapolated to the total German population, this is approximately 20 incident TBI‐aHO cases per year. The estimated and extrapolated 2022 prevalence is 520 cases in Germany (95% CI 460–590). Space‐occupying lesion, or lesions that expand and displace normal neural structures, is the primary diagnosis leading to aHO in patients with TBI (TBI‐aHO), accounting for almost 50% of all identified patients with TBI‐aHO (Figure S2). General practitioners are involved in the diagnosis of obesity and AVP‐D after the index event. Endocrinologists are less commonly involved, and, if involved, it is in the diagnosis of hypopituitarism in our study cohort.

**TABLE 1:** Characteristics of patients with aHO in a population with TBI and UM.

Population	Observed cases in study database	aHO incidence rate (/1,000,000)	Mean age, years (SD)	Age groups, years			Female
				< 20	20–64	≥ 65	
TBI‐aHO	11	1.78	42 (15)	*n* < 5	73%	*n* < 5	*n* < 5
UM‐aHO	14	2.12	37 (18)	*n* < 5	73%	*n* < 5	55%

*Note*: Information with *n* < 5 cases is censored due to data protection reasons.

Abbreviations: aHO, acquired hypothalamic obesity; SD, standard deviation; TBI, traumatic brain injury; UM, unspecified microinjury.

**FIGURE 3:**
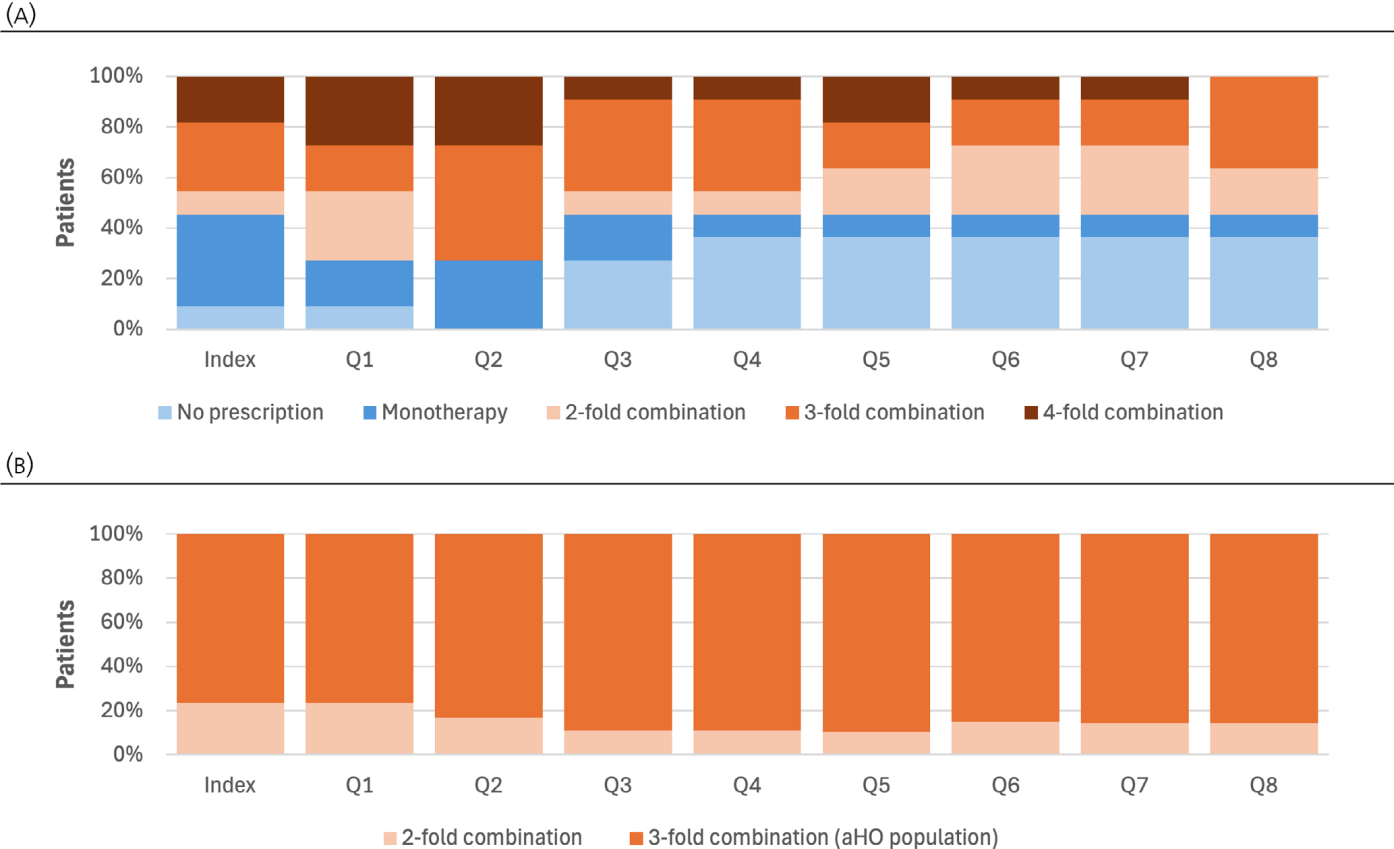
Neuroendocrine treatment combinations in patients with (A) TBI‐aHO and (B) UM‐aHO. Neuroendocrine treatment combinations were among inclusion criteria of patients with UM‐aHO, thus no patients without a prescription or with monotherapy are included. aHO, acquired hypothalamic obesity; Q, quarter; TBI, traumatic brain injury; UM, unspecified microinjury.

Patients with TBI‐aHO receive extensive pharmacotherapies for neuroendocrine deficits. In addition to desmopressin (a criterion for aHO validation, therefore forcing 100% of patients with a prescription in the follow‐up period), patients frequently receive hydrocortisone or levothyroxine‐sodium (Table 2). Among patients with validated aHO, 18% received somatropin replacement therapy during follow‐up, indicating relevant growth hormone (GH)–insulin‐like growth factor‐ 1 axis involvement. Observed specific medications are also frequently used in combination. For example, at the second quarter after TBI incidence, 72% of all identified patients with TBI‐aHO received a three‐fold combination of neuroendocrine treatments (Figure 3A). From the third quarter onwards, at least 55% of our population with TBI‐aHO received a permanent triple drug combination.

**TABLE 2:** Share of patients (%) with aHO with neuroendocrine treatments in the post‐observational period.

Population	Prescription drug	Index	Post‐observational period								Total
			Q1	Q2	Q3	Q4	Q5	Q6	Q7	Q8	
TBI‐aHO (*n* = 11)	Desmopressin	82	91	100	73	55	55	55	55	64	100
	Hydrocortisone	55	64	73	45	55	55	55	55	55	73
	Levothyroxine‐sodium	55	45	64	45	45	45	36	36	36	73
	Somatropin	9	0	9	0	9	18	9	9	0	18
	Testosterone	9	36	27	18	9	0	0	0	0	45
UM‐aHO (*n* = 14)	Desmopressin	59	68	59	68	59	68	55	73	64	100
	Hydrocortisone	45	55	55	50	45	59	55	55	50	82
	Levothyroxine‐sodium	45	55	64	59	64	50	86	73	59	86
	Somatropin	0	0	5	5	14	9	18	9	14	27
	Testosterone	9	14	18	14	14	9	18	18	14	23

Abbreviations: aHO, acquired hypothalamic obesity; Q, quarter; TBI, traumatic brain injury; UM, unspecified microinjury.

### 3.3 aHO in patients with UM

After excluding patients with tumor diagnoses and treatment, 20 patients were validated as patients with UM‐aHO. Due to the uncertainty associated with the incidence of the index date, the initiation of two endocrine substitution therapies within 1 year before or after the incident obesity is also used as an additional criterion for UM‐aHO. Of patients identified with incident obesity diagnosis and incident AVP‐D, 70% (*n* = 14) of patients fulfilled the initiation of two additional endocrine substitution therapies. These patients are, on average, younger (34 years) than patients with TBI‐aHO and a higher proportion are female (55%) (Table 1).

The estimated incidence of UM‐aHO is 2.12 cases per 1,000,000 persons. Extrapolated to the total German population, this is ~28 incident cases with UM‐aHO per year. The estimated and extrapolated 2022 prevalence is 660 cases in Germany (95% CI 585–750).

Similar to the TBI‐aHO patients, the UM‐aHO patients received extensive neuroendocrine therapies during our observation period. In addition to desmopressin, four of five patients also received hydrocortisone or levothyroxine‐sodium within 2 years after the index date (Table 2). In total, 27% of patients received GH replacement therapy with somatropin during follow‐up, supporting hypothalamic–pituitary axis dysfunction as a potential driver of aHO. The pharmacological treatment burden is further reflected by the fact that only a small number of additional patients would qualify as likely UM‐aHO cases if the definition were limited to those receiving dual combination therapy including desmopressin (Figure 3B). In the UM‐aHO validation process, 44% of validated patients were initiated on a three‐fold neuroendocrine therapy combination within 1 year prior to the incident UM diagnosis, with 57% receiving this combination therapy at the index date or within the following year (Figure S3).

### 3.4 Sensitivity analyses

The most critical source of uncertainty relates to the identification of cases with aHO within the cohort with UM, particularly ensuring the exclusion of false‐positive classifications. To mitigate this, we applied a 2‐year washout period for incident obesity among patients with UM, which aimed to rule out the development of general obesity prior to cohort entry. This adjustment had no effect on identified patient numbers. When modifying the treatment criterion—considering desmopressin in combination with at least one (instead of two) endocrine substitution therapies—the impact was minimal, leading to the inclusion of only two additional cases.

In both the cohorts with UM and TBI, we also assessed the sensitivity of aHO incidence estimates by including patients with prevalent obesity in the aHO case definition. This broader definition did not result in any major changes to incidence estimates.

### 3.5 Additional findings

As part of the sensitivity analysis, *n* < 5 additional patients were identified who met the predefined diagnostic and validation criteria for aHO. Unlike in the UM cohort, however, a specific structural defect could be determined for these patients, marking the onset of both the weight trajectory and the need for neuroendocrine replacement therapy. In one case, this event was the diagnosis of septo‐optic dysplasia (SOD; ICD‐10‐GM Q04.4); in the other, a diagnosis of a midline brain structure disorder (ICD‐10‐GM G93.‐). Due to the small number of cases, these patients were not included in the primary analysis.

## 4 DISCUSSION

aHO epidemiology is poorly understood because incidence and prevalence are related to several very rare underlying conditions. In a previous study, we expanded the spectrum of TTR‐aHO cases beyond patients with craniopharyngioma. This analysis identified cases with TTR‐aHO related to all tumor entities described in the data from the patient self‐reported data in the International Registry of Hypothalamic Obesity Disorders (IRHOD).^8^ The study found that, while craniopharyngioma is the most researched etiology of aHO, patients with other tumors, such as benign neoplasm of the pituitary gland, may also develop aHO. We also found that additional validation by a desmopressin prescription—as a confirmation of AVP‐D—had a significant effect on the number of patients with aHO.^24^ This validation is important, as AVP‐D is associated with HO because vasopressin is produced in the hypothalamus, transported via the pituitary stalk to the posterior lobe of the pituitary gland, and released into the bloodstream.^25^

The present research focuses on the incidence and prevalence of aHO following other trauma to the hypothalamus; specifically, TBI and other UM to the hypothalamic–pituitary axis. Although previous evidence links TBI to neuroendocrine dysfunction^10^ and weight gain,^13^ this research provides valuable first insights into the epidemiology of aHO beyond tumor‐related etiologies. In this and our previous real‐world data analyses, we identified a total of 62 incident cases of aHO between 2010 and 2020 after exclusion of duplicates (or approximately 2500 prevalent cases in 2019–2020). Of these, 60% were attributable to tumor diagnoses and associated treatments, 22% to UM, and 18% to TBI. These findings provide insight into the relative contribution of distinct etiological pathways leading to aHO. However, this distribution does not necessarily reflect the overall epidemiology of aHO in the broader population. Case identification was based on a predefined algorithm, which may have introduced selection bias toward more clearly coded etiologies. In addition, two further cases—one with SOD and one with a midline brain structure disorder—were identified through manual review of the study cohort. Given the absence of specific diagnostic codes for aHO in routine data, further undetected cases cannot be ruled out. This highlights the need for improved diagnostic coding practices and algorithm refinement to ensure comprehensive capture of the full clinical spectrum of aHO.

We found that the profile of aHO in patients with TBI and UM is consistent with those of better‐known etiologies such as craniopharyngioma and other tumors. Similar to aHO in other contexts, incident obesity and AVP‐D occur rapidly after the TBI index date and patients show a high need for neuroendocrine replacement therapy shortly following the index date. Our results are in line with the suggestion that the prescription of desmopressin might be an important early identifier of patients with aHO. This is important, as the early development of TBI‐aHO may go unnoticed while patients, families, and medical teams focus on the acute management of TBI; or, for patients with UM, the diagnosis of AVP‐D and desmopressin prescription could be the first sign of serious hypothalamic dysfunction and underlying damage. Early recognition of the risk for aHO is necessary for prevention and intervention before substantial weight gain occurs.^26^, ^27^ Moreover, the TBI‐aHO and UM‐aHO cases identified in this study are largely comparable to TTR‐aHO patients in terms of demographic characteristics. The mean age at aHO incidence ranges from 37 years (standard deviation [SD] 18) to 42 years (SD 15) across groups. While two‐thirds of TTR‐aHO patients are female, the majority of TBI‐aHO patients (73%) are male. In contrast, the UM‐aHO group shows a balanced gender distribution.

Beyond trauma‐ and tumor‐related etiologies, other forms of hypothalamic–pituitary axis injury may contribute to aHO. One plausible mechanism is postpartum pituitary necrosis (Sheehan syndrome). In exploratory, aggregate‐level analyses, patterns were compatible with this hypothesis; however, due to data‐protection obligations and the anonymized nature of claims data, patient‐level linkages cannot be disclosed. The biological overlap—combining anterior pituitary deficits (GH, prolactin) and, rarely, AVP‐D—supports its inclusion among plausible pathways to aHO. Future research using refined diagnostic codes for postpartum lactation failure or hypoprolactinemia and prospective endocrine validation is warranted.

Our results highlight the intensified pharmacological management of neuroendocrine disorders, characterized by the concurrent use of multiple pharmacological agents—a treatment pattern consistently observed in aHO regardless of underlying etiology. For example, 45% of patients with TBI‐aHO received desmopressin and two other neuroendocrine replacements within two quarters of the index date, and 41% of patients with UM‐aHO initiated neuroendocrine therapy within 1 year of the index date. Given that available treatments are known to have limited efficacy, and pharmaceutical agents for obesity treatment are associated with considerable side effects, new treatment options for aHO are currently under development. Setmelanotide, an MC4R agonist, has demonstrated strong efficacy in aHO in a large double‐blind, randomized controlled phase 3 trial,^28^ following robust phase‐2 trial data showing a statistically significant proportion of tumor patients achieving ≥5% reduction in body mass index [BMI].^29^ In the context of an emerging targeted and effective therapy, timely and accurate diagnosis of aHO becomes even more critical to ensure that eligible patients can benefit from disease‐modifying treatment.

While the current study applied validated claims‐based criteria to identify and confirm cases of aHO, future analyses may benefit from incorporating negative control groups to further assess the specificity of the applied case definition. For example, the inclusion of patients with AVP‐D and desmopressin therapy without concurrent or subsequent obesity diagnoses could serve as a reference population to better contextualize treatment patterns and evaluate potential misclassification bias. Such an approach may help to clarify whether the identified associations between endocrine dysfunction and obesity are unique to hypothalamic injury or could reflect broader treatment indications. Although not required for the objectives of this initial epidemiological exploration, a negative control framework could be valuable in refining future algorithm development and strengthening causal inference.

Given the heterogeneity and diagnostic uncertainty within the UM‐aHO cohort, future research may also consider applying latent class analysis (LCA) to further validate and stratify patient subgroups based on patterns of endocrine deficits, treatment intensity, and diagnostic constellations. As UM‐aHO cases lack a clear index event, LCA could help distinguish between high‐ and low‐probability aHO phenotypes based on shared, but indirectly observed, characteristics. This approach would allow for the identification of latent patient classes that may differ in etiology, severity, and prognosis –thereby refining epidemiological estimates and improving algorithmic specificity. Incorporating LCA could also facilitate the development of data‐driven risk scores to support early clinical recognition of aHO in the absence of overt traumatic or tumorous insults.

The dataset represents one of this study's primary strengths. The large cohort size leads to a relatively high number of patients with aHO in the considered underlying conditions and provides a comprehensive database for the validation of TBI‐aHO and UM‐aHO through incident obesity, AVP‐D diagnosis, and desmopressin prescription. The large dataset allows us to observe relevant clinical patient constellations that may not have been possible based on case reports.

This study is subject to limitations inherent to German routine data, which lack clinical information such as laboratory values, anthropometric measures (e.g., BMI), MRI results, and inpatient specialist contacts. Consequently, the assessment of weight gain is constrained by the reliance on ICD‐10 codes for obesity, which are known to have limited sensitivity.^30^ Nonetheless, the clinical relevance of rapid weight gain in aHO may have led to more accurate documentation in this context. Yet, excessive glucocorticoid replacement doses could have contributed to weight gain in some patients, representing an additional limitation. Additionally, information regarding the specific cause or context of TBI (e.g., sports‐related, road accidents, or military trauma) is not documented in the claims database. While a wide range of diagnostic codes for TBI was used for case identification (see Table S1), the limited number of validated aHO cases precluded the derivation of robust associations with specific TBI subtypes. Finally, the attribution of prescriptions to specific indications is challenging in claims data, particularly in multimorbid patients. As a result, desmopressin use for enuresis cannot be excluded entirely, though such use is uncommon and limited primarily to pediatric populations. The study design and methods face other general limitations as well. First, there is no available information on the history of aHO‐applied definition and validation criteria prior to the observational period for defining incident cases. If, for example, a patient with TBI‐aHO had a 2‐year interval between TBI‐related hospitalizations, a 1‐year washout period would be too short, and the prevalent case would be misclassified as an incident case. Second, self‐selection of patients is possible; for example, patients with severe weight gain might be more likely to seek treatment after TBI or other related conditions. However, in the German setting, all patients are monitored closely in follow‐up visits after congenital defects or traumatic injuries. In addition, the ubiquity of associated neuroendocrine disorders and their treatment implies that all patients are likely to be under active medical care during this period. In this study, we focus on the analysis of aHO specifically, whereas future research should examine the epidemiology of other aHO diseases, such as systemic non‐tumorous disorders like sarcoidosis and hypophysitis, or the sequelae of HO of different etiology. Another limitation is the lack of information on the specific UM cohort definitions, which prevents a definitive assessment of whether observed effects are transient or persistent. Moreover, the 2‐year observation period limits conclusions regarding the long‐term durability of hormone replacement therapy effects.

## 5 CONCLUSIONS

This is the first real‐world database analysis of the epidemiology of aHO in patients with non‐tumor–related conditions. Analysis of the epidemiology of rare diseases with aHO is extremely important not only to understand the diseases, but also to inform clinical and health‐economic decision‐making. Due to the lack of defining standards for clinical documentation, epidemiological estimation is associated with uncertainty.

## AUTHOR CONTRIBUTIONS

**Julian Witte:** Conceptualization; methodology; data curation; investigation; funding acquisition; writing – original draft; visualization; project administration. **Nicolas Touchot:** Conceptualization; methodology; funding acquisition; writing – review and editing. **Manuel Batram:** Methodology; data curation; writing – review and editing; supervision. **Jana Diekmannshemke:** Methodology; data curation; formal analysis; writing – review and editing. **Mathias Flume:** Conceptualization; methodology; writing – review and editing. **Hermann L. Müller:** Methodology; writing – review and editing; validation.

## FUNDING INFORMATION

Rhythm Pharmaceuticals funded this study and was involved in the development of the data analysis strategy but neither had access to the database nor conducted the data analysis. Rhythm Pharmaceuticals also took charge of all costs associated with the development of this manuscript.

## CONFLICT OF INTEREST STATEMENT

J.W. and M.B. own shares in Vandage GmbH. J.D. is employed at Vandage. Vandage received funding from Rhythm Pharmaceuticals to perform the study related to this manuscript. Vandage received payments from Rhythm, Alexion, AstraZeneca, BMS, GSK, Janssen, Moderna, MSD, Pfizer, PTC Therapeutics, Sanofi Pasteur, Seqirus, Viatris and consulting fees and grants from AOK Rheinland/Hamburg, BARMER, DAK‐Gesundheit, German G‐BA, and Techniker Krankenkasse. H.L.M. has received reimbursement of participation fees for scientific meetings and continuing medical education events from the following companies: Ferring, Lilly, Pfizer, Sandoz/Hexal, Novo Nordisk, IPSEN, Rhythm Pharmaceuticals, and Merck Serono; he has received reimbursement of travel expenses from IPSEN and Rhythm Pharmaceuticals and lecture honoraria from Pfizer. N.T. is employed by Rhythm Pharmaceuticals. M.F. owns GeneAccess GmbH (GA). GA received consulting fees from Rhythm Pharmaceuticals and AstraZeneca.

## ETHICS STATEMENT

As the study did not gather patient or individual‐level data or involve any interventions, informed consent was not applicable. Positive ethics vote was granted from the Medical Association of Westphalia‐Lippe Ethics Commission (#2024‐616‐f‐N).

## Supporting information


**Figure S1.** Timeframes of data usage for patient inclusion and follow‐up. aHO, acquired hypothalamic obesity; TBI, traumatic brain injury; UM, unspecified microinjury.

**Figure S2.** Diagnoses defining the underlying conditions in patients with TBI‐aHO. Diagnosis of two or more entities per patient possible.

**Figure S3.** Patients with UM‐aHO initiating neuroendocrine drug combination within 1 year before or after index date.

**Table S1.** Study cohort definitions.
**Table S2.** Drug prescriptions evaluated in an TBI‐aHO and UM‐aHO cohort.

## Data Availability

The data that support the findings of this study are available from GWQ‐ServicePlus AG. Restrictions apply to the availability of these data, which were used under license for this study. Data are available from the author(s) with the permission of GWQ‐ServicePlus AG.
