# Supplementary figures and images for: Epidemiology of acquired hypothalamic obesity following traumatic brain injury and nonspecific hypothalamic microinjury: A nationwide German claims data analysis
Source: J Neuroendocrinol. 2025 Nov 14;38(1):e70108. doi: 10.1111/jne.70108 (PMC12799323; doi:10.1111/jne.70108)

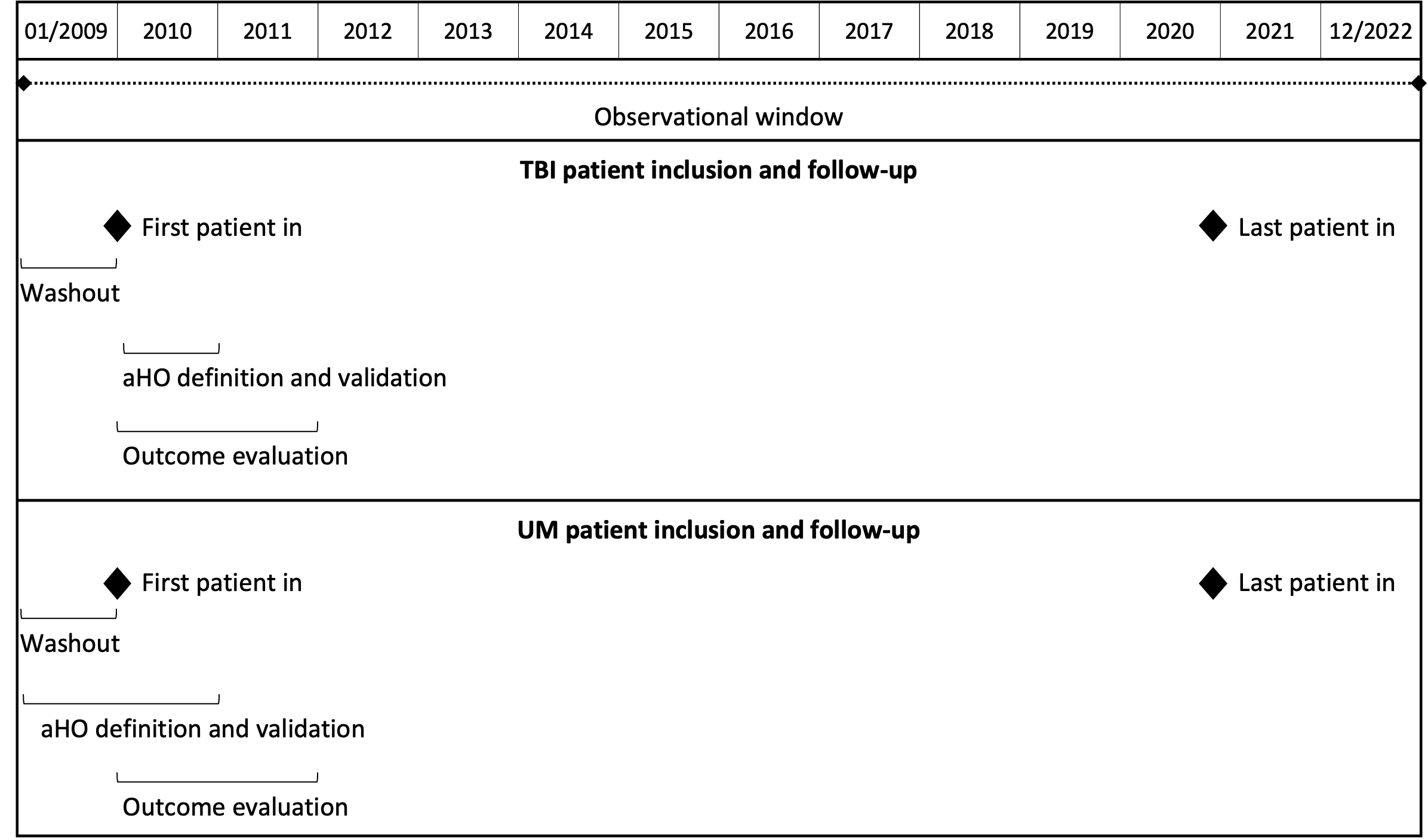

Supplement: Supplementary file 1 — Figure S1. Timeframes of data usage for patient inclusion and follow‐up. aHO, acquired hypothalamic obesity; TBI, traumatic brain injury; UM, unspecified microinjury. [file JNE-38-e70108-s004.png]

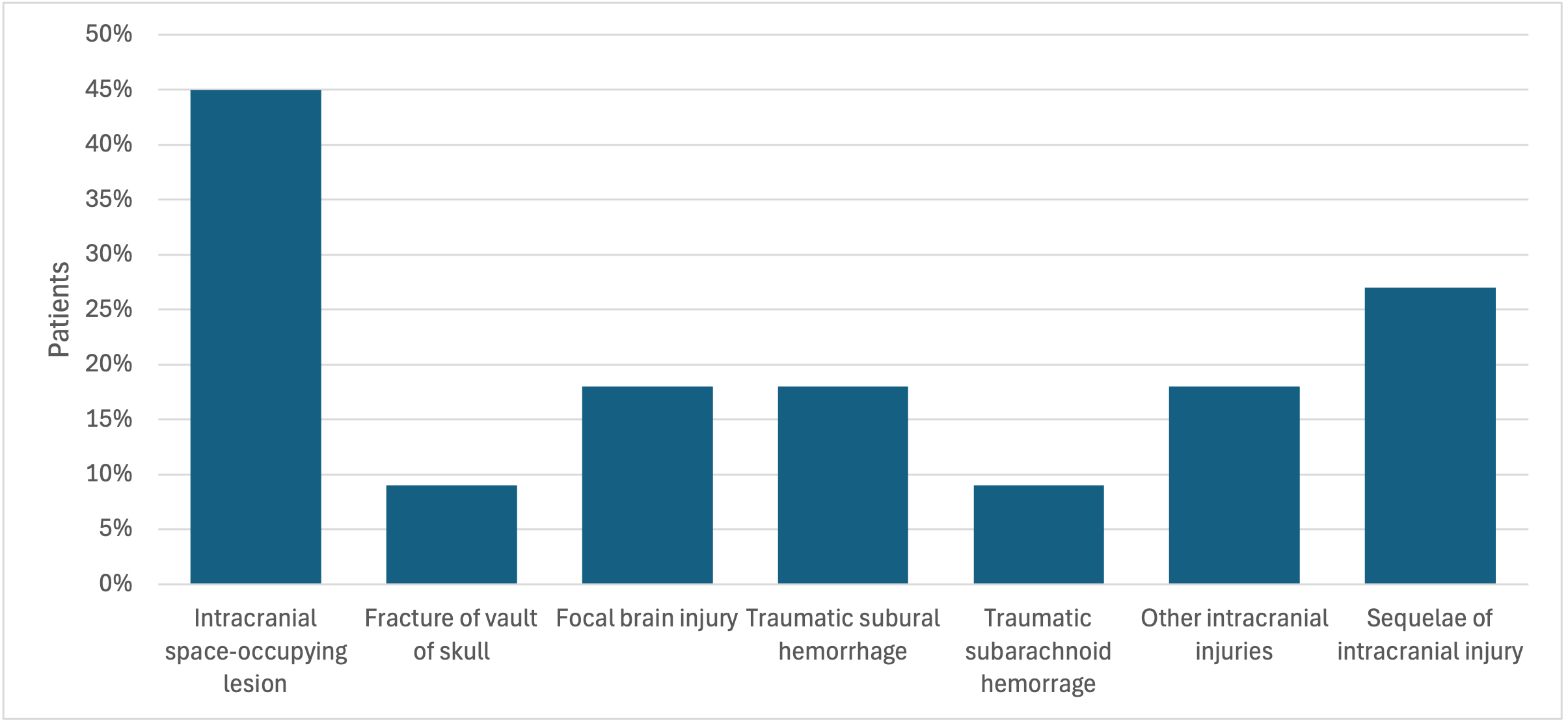

Supplement: Supplementary file 2 — Figure S2. Diagnoses defining the underlying conditions in patients with TBI‐aHO. Diagnosis of two or more entities per patient possible. [file JNE-38-e70108-s003.png]

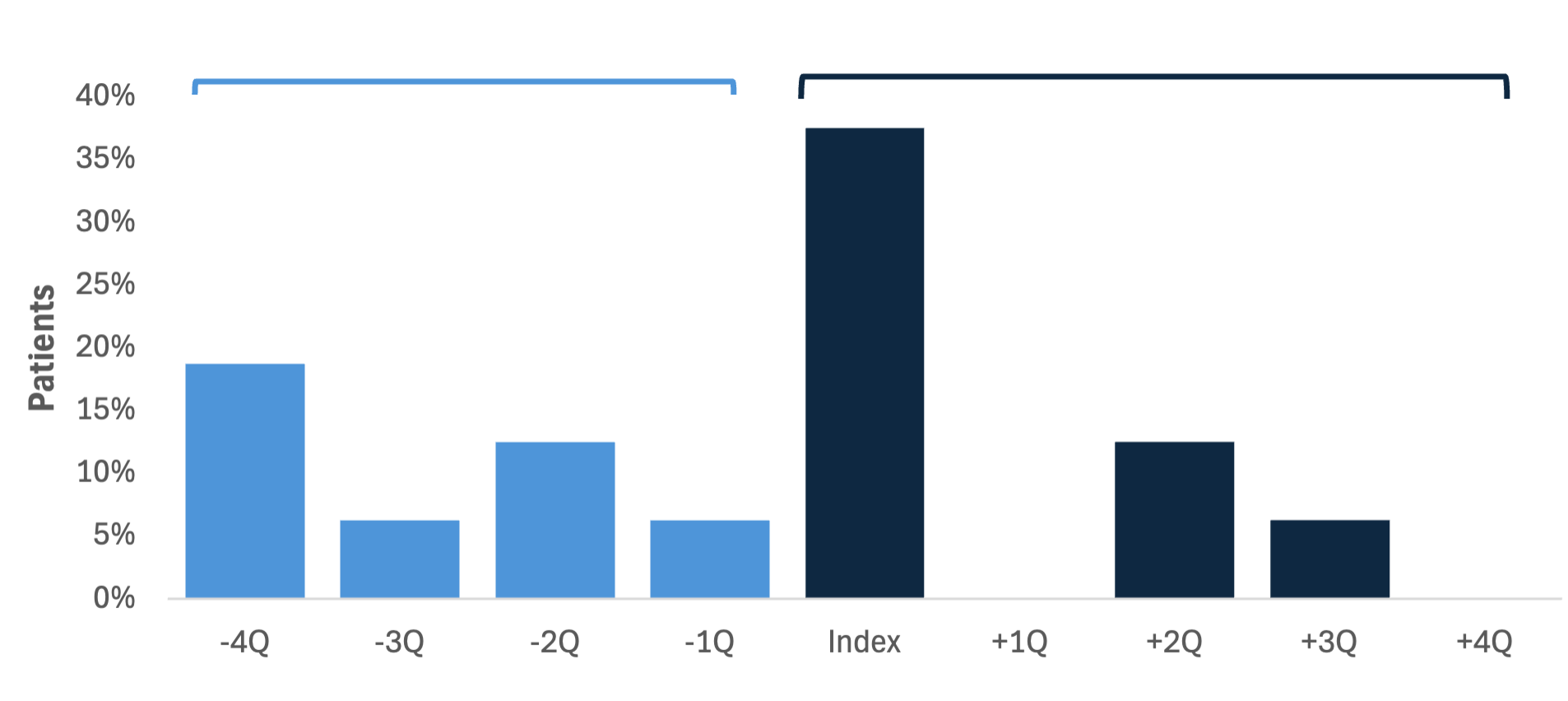

Supplement: Supplementary file 3 — Figure S3. Patients with UM‐aHO initiating neuroendocrine drug combination within 1 year before or after index date. [file JNE-38-e70108-s002.png]
