# Supplementary material for: Epidemiology of acquired hypothalamic obesity following traumatic brain injury and nonspecific hypothalamic microinjury: A nationwide German claims data analysis
Source: J Neuroendocrinol. 2025 Nov 14;38(1):e70108. doi: 10.1111/jne.70108 (PMC12799323; doi:10.1111/jne.70108)
Supplement: Supplementary file 4 — Table S1. Study cohort definitions. Table S2. Drug prescriptions evaluated in an TBI‐aHO and UM‐aHO cohort. [file JNE-38-e70108-s001.docx]

**Supplemental material**

**Table S1: Study cohort definitions**

| **#** | **ICD-10-GM** | **Definition** | **Case definition and validation** |
| --- | --- | --- | --- |
| **Population with traumatic brain injury** | | | |
| 1 | S02.0 | Fracture of vault of skull | Diagnosis frequency: [min1], Combination with following diagnoses: [OR] |
| 2 | S02.1 | Fracture of base of skull | [min1], [OR] |
| 3 | S02.7 | Multiple fractures involving skull and facial bones | [min1], [OR] |
| 4 | S09.7 | Multiple injuries of head | [min1], [OR] |
| 5 | T90.2 | Sequelae of fracture of skull and facial bones | [min1], [OR] |
| 6 | T90.5 | Sequelae of intracranial injury | [min1], [OR] |
| 7 | R90.0 | Intracranial space-occupying lesion | [min1], [OR] |
| 8 | S06 | Intracranial injury, excluding "concussion" (S06.0) | [min1], [OR] |
| 9 | S06.5 | Traumatic subdural hemorrhage | [min1], [OR] |
| 10 | S06.6 | Traumatic subarachnoid hemorrhage | [min1], [OR] |
| 11 | S06.33 | Focal cerebral hematoma | [min1], [OR] |
| 12 | S06.9 | Intracranial injury, unspecified | [min1], [OR] |
| 13 | S06.8 | Other intracranial injuries | [min1], [OR] |
| 14 | S06.4 | Epidural hemorrhage | [min1], [OR] |
| 15 | S06.21 | Diffuse cerebral contusion | [min1], [OR] |
| 16 | S06.31 | Focal cerebral contusion | [min1], [OR] |
| 17 | S06.23 | Multiple intracerebral and cerebellar hematomas | [min1], [OR] |
| 18 | S06.30 | Focal cerebral and cerebellar injury, unspecified | [min1], [OR] |
| 19 | S06.1 | Traumatic cerebral oedema | [min1], [OR] |
| 20 | S06.34 | Focal cerebellar hematoma | [min1], [OR] |
| 21 | S06.20 | Diffuse cerebral and cerebellar injury, unspecified | [min1], [OR] |
| 22 | S06.28 | Other diffuse cerebral and cerebellar injuries | [min1], [OR] |
| 23 | S06.38 | Other focal cerebral and cerebellar injuries | [min1], [OR] |
| **Population with unspecified microinjury** | | | |
| 24 | H47.0 | Disorders of optic nerve, not elsewhere classified | [min1], [AND one of #27-#31] |
| 25 | E23.0 | Hypopituitarism | [min1], [AND one of #27-#31] |
| 26 | E23.6 | Other disorders of pituitary gland | [min1], [AND one of #27-#31] |
| 27 | LANR 38, 44 | 1 Contact with a pediatric neurologist | [min1], [AND one of #24-#26] |
| 28 | ATC H01AC01 | 1 Prescription of somatropin | [min1], [AND one of #24-#26] |
| 29 | H55.- | 1 Diagnosis of nystagmus | [min1], [AND one of #24-#26] |
| 30 | E34.4, Q87.1 | 1 Diagnosis of reduced growth rate | [min1], [AND one of #24-#26] |
| 31 | E16.1, E16.2, ATC H03AA01 | 1 Diagnosis of a rare neuroendocrine dysfunction | [min1], [AND one of #24-#26] |

**Table S2: Drug prescriptions evaluated in an TBI-aHO and UM-aHO cohort**

| **Drug** | **ATC-Code** | **Case definition and validation** |
| --- | --- | --- |
| Desmopressin | H01BA02 | [min1] |
| Hydrocortisone | H02AB09 | [min1] |
| Levothyroxine-sodium | H03AA01 | [min1] |
| Somatropin | H01AC01 | [min1] |
| Testosterone | G03BA03 | [min1] |
| Estrogens | G03C | [min1] |
| Progestogens | G03D | [min1] |
| Female hormone combination treatments | G03F | [min1] |

**[Figure S1]**

**[Figure S2]**

**[Figure S3]**
